# NMR Structure and Biophysical Characterization of Thermophilic Single-Stranded DNA Binding Protein from *Sulfolobus Solfataricus*

**DOI:** 10.3390/ijms23063099

**Published:** 2022-03-13

**Authors:** Min June Yang, Jinwoo Kim, Yeongjoon Lee, Woonghee Lee, Chin-Ju Park

**Affiliations:** 1 Department of Chemistry, Gwangju Institute of Science and Technology, Gwangju 61005, Korea; minjune1590@gm.gist.ac.kr (M.J.Y.); wlsdn8810@gist.ac.kr (J.K.); 2 Department of Chemistry, University of Colorado Denver, Denver, CO 80217-3364, USA; bioyj1012@gmail.com

**Keywords:** thermophile, thermostability, high temperature, OB-fold, single-stranded binding, NMR, backbone relaxation, solution structure, chemical shift perturbation, *Sulfolobus solfataricus*

## Abstract

Proteins from *Sulfolobus solfataricus (S. solfataricus)*, an extremophile, are active even at high temperatures. The single-stranded DNA (ssDNA) binding protein of *S. solfataricus* (SsoSSB) is overexpressed to protect ssDNA during DNA metabolism. Although SsoSSB has the potential to be applied in various areas, its structural and ssDNA binding properties at high temperatures have not been studied. We present the solution structure, backbone dynamics, and ssDNA binding properties of SsoSSB at 50 °C. The overall structure is consistent with the structures previously studied at room temperature. However, the loop between the first two β sheets, which is flexible and is expected to undergo conformational change upon ssDNA binding, shows a difference from the ssDNA bound structure. The ssDNA binding ability was maintained at high temperature, but different interactions were observed depending on the temperature. Backbone dynamics at high temperature showed that the rigidity of the structured region was well maintained. The investigation of an N-terminal deletion mutant revealed that it is important for maintaining thermostability, structure, and ssDNA binding ability. The structural and dynamic properties of SsoSSB observed at high temperature can provide information on the behavior of proteins in thermophiles at the molecular level and guide the development of new experimental techniques.

## 1. Introduction

*Sulfolobus* is one of the well-known hyperthermophilic archaebacterial genera [1]. Unlike mesophilic eukaryotes and bacteria, which are sensitive to external conditions, *Sulfolobus* can survive at extremely low pH or high temperature [1,2,3,4,5]. *Sulfolobus solfataricus (S. solfataricus)* is adapted to high temperature via lipid composition changes [6], protection of its DNA with DNA binding proteins [7], and expression of a unique DNA topoisomerase [8]. Because they have the ability to survive in such harsh conditions, proteins from *S. solfataricus* are widely used in biological experiments and industrial applications that require low pH or high-temperature conditions [5]. Glyceraldehyde phosphate dehydrogenase, carboxypeptidase, alanine: glyoxylate transaminase, γ-lactamase, and other enzymes of *S. solfataricus* have been applied as industrial biocatalysts [9].

Single-stranded DNA (ssDNA) binding proteins (SSBs) of *S. solfataricus* are also used in biotechnological applications under harsh conditions. SSBs are proteins that bind to ssDNA non-sequence specifically. During the DNA replication or repair process, they prevent ssDNAs released by a helicase from returning to double-stranded DNAs (dsDNAs), and thereby increase the DNA polymerase activity. Thus, SSBs are essential for all living organisms to preserve their genomes [10,11]. At high temperatures, dsDNAs substantially melt into ssDNAs, and ssDNAs are much more vulnerable to damage than dsDNAs [12]. Therefore, hyperthermophilic archaebacterial species, including *S. solfataricus*, that inhabit extremely hot environments, overexpress SSBs to protect single-stranded nucleic acids from severe conditions and retain their genes [13].

**Figure 1:**
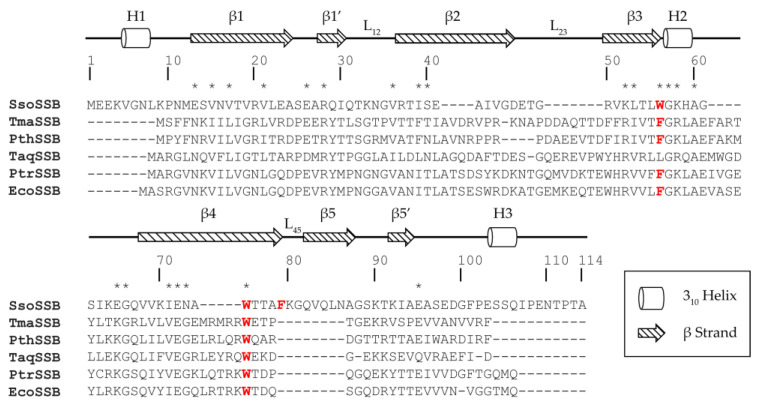
Sequence alignment of SsoSSB with SSBs from various bacteria: hyperthermophilic *Thermotoga maritima* (TmaSSB) and *Pseudothermotoga thermarum* (PthSSB); thermophilic *Thermus aquaticus* (TaqSSB), and *Pseudoalteromonas translucida* (PtrSSB); mesophilic *Escherichia coli* (EcoSSB). Conserved residues are indicated with an asterisk. Aromatic residues participating in base-stacking upon DNA binding are colored red. The secondary structure of SsoSSB from UniProt [21] and a previous study [14] are depicted above the sequence.

*S. solfataricus* SSB (SsoSSB) consists of 148 amino acids. The unbound structure determined using X-ray crystallography (PDB ID: 1O7I [14]) and the ssDNA bound structure determined at 25 °C using nuclear magnetic resonance (NMR) spectroscopy (PDB ID: 2MNA [15]) showed that the protein has a well-conserved oligonucleotide/oligosaccharide binding fold (OB-fold) domain. OB-folds consist of a well-conserved β barrel structure with five β strands capped by one α helix and an ssDNA binding pocket composed of L_12_ and L_45_ loops [16,17]. Human replication protein A (hRPA) [18], *Escherichia coli* SSB [19], and human mitochondrial SSB [20] are examples of SSBs that can bind to ssDNA strongly. Unlike other well-characterized SSBs, in which two aromatic residues are conserved and are important for ssDNA binding, SsoSSB has an extra aromatic residue (Figure 1), which increases the binding affinity for ssDNA by forming an additional π-π stacking interaction with ssDNA [13,14,15]. Although the structural properties of SsoSSB have been reported at room temperature, it remains unclear which structural and dynamic features are important for its high thermostability and ssDNA binding properties at the optimal survival temperature (55 to 88 °C [1,2,3,4,5]).

Here, we determined the structure of SsoSSB at high temperature (50 °C) using NMR spectroscopy. SsoSSB was shown to maintain a well-conserved OB-fold structure even at this high temperature, and its thermostability was measured using differential scanning calorimetry (DSC) and circular dichroism (CD) spectroscopy. The ssDNA binding activity and backbone dynamics of the protein were also investigated at high temperature using NMR spectroscopy. The spin relaxation experiments revealed that the protein surprisingly retained a highly rigid structure even at high temperature. Moreover, the protein was still able to interact with ssDNA at elevated temperatures. To determine the role of the N-terminal region in the thermostability and DNA binding of the protein, we analyzed the properties of an N-terminal deletion mutant using DSC, CD spectroscopy, isothermal titration calorimetry (ITC), and NMR. Our findings provide important understanding of thermophilic SsoSSB near its physiological conditions and fundamental insights into its potential for biotechnological applications in high-temperature conditions.

## 2. Results

### 2.1. Thermostability of SsoSSB

SSBs have highly conserved sequences ranging from mesophiles to hyperthermophiles (Figure 1). Unlike other SSBs, SsoSSB has a unique N-terminal region containing an additional helix H1. All the individual secondary structure elements other than H1 and β5 combine to form the well-conserved OB-fold structure. Compared with the canonical OB-fold structures, SsoSSB showed unique secondary structures (H1, β1’, and β5’) [14]. In addition to the highly conserved aromatic residues, W56 and W75, SsoSSB possesses an additional F79, which contributes to the increase in binding affinity for ssDNA by forming an additional base-stacking interaction [14].

**Figure 2:**
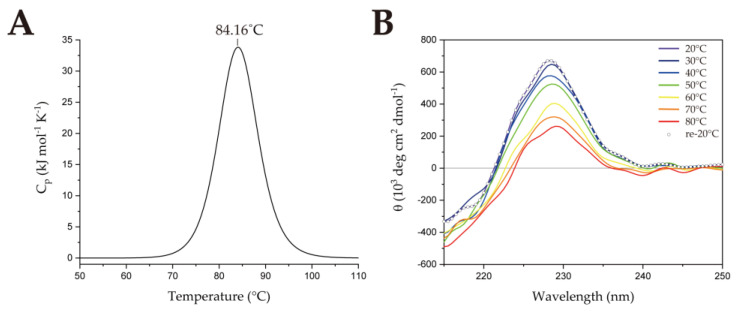
Thermostability of SsoSSB_1–114_. (**A**) Melting temperature (T_m_) of SsoSSB_1–114_ measured by differential scanning calorimetry. (**B**) Circular dichroism spectroscopy was used to investigate the effect of temperature on the secondary structure of SsoSSB_1–114_.

To compare the thermostability of SsoSSB with other thermophilic SSBs, we performed DSC. From the DSC data, the melting temperature (T_m_) of SsoSSB_1−114_ was measured at 84.16 °C (Figure 2a), which is lower than that of the hyperthermophilic TmaSSB (109.3 °C) [22]. CD spectroscopy was performed at 20 °C to confirm the secondary structure of the protein (Figure S1a). There is a negative peak at 215 nm, which comes from β strands [23], and a positive peak at 228 nm, which indicates β-II type β-rich protein [24,25]. We also monitored structural changes over the temperature range of 20 to 80 °C using CD. Molar ellipticity at 228 nm was plotted at intervals of 2 °C (Figure S1b). As the temperature increased from 20 to 80 °C, the molar ellipticity at 228 nm decreased by about 452,000 deg cm^2^ dmol^−1^. This suggests that the secondary structure became destabilized but not completely denatured [23,24,25,26,27]. When the temperature reached 80 °C, the sample was cooled to 20 °C to confirm whether SsoSSB_1–114_ refolded after heating (Figure 2b). Our result showed that the CD spectrum was fully recovered, which implies that denaturation of the protein is reversible. This is consistent with the previous studies showing that the protein was not fully denatured, even at high temperatures, using ^1^H-^15^N heteronuclear single-quantum coherence (HSQC) spectra [15,28].

### 2.2. Solution Structure of SsoSSB_1–114_ at High Temperature

We previously reported the backbone and sidechain atom chemical shift assignments of the protein at 50 °C (BMRB 50523) and presented 2D ^1^H-^15^N HSQC spectra with the assignment [28]. To obtain high temperature distance constraints, nuclear Overhauser effects (NOEs) were observed from ^15^N- and ^13^C-edited-NOESY-HSQC experiments performed at 50 °C. The AUDANA algorithm [29] generated distance and torsion angle constraints using the protein sequence, chemical shift assignments, and NOESY data as inputs. TALOS-N [30] and Xplor-NIH [31] operations were automated by AUDANA for torsion angle constraints and structure calculations, respectively.

We obtained 893 distance constraints from the NOESY data and 193 angle constraints from TALOS-N. In the previous study of the NMR structure at room temperature, 2294 intramolecular constraints were used for protein structure calculation [15]. It is known from previous studies that fewer constraints are measured at higher temperatures [32,33] because of various factors, including the partial denaturation of secondary structures and the reduced sensitivity at elevated temperatures. It was also found that there were relatively small numbers of medium-range constraints (85, 9.9% of total distance restraints) because the protein is mainly composed of β strands with a small amount of 3_10_ helical structure. For the structure calculation at room temperature, a similar proportion of medium-range restraints (8.5% of total distance restraints) was used [15]. The number of NOE constraints for each residue is shown in Figure S2.

**Figure 3:**
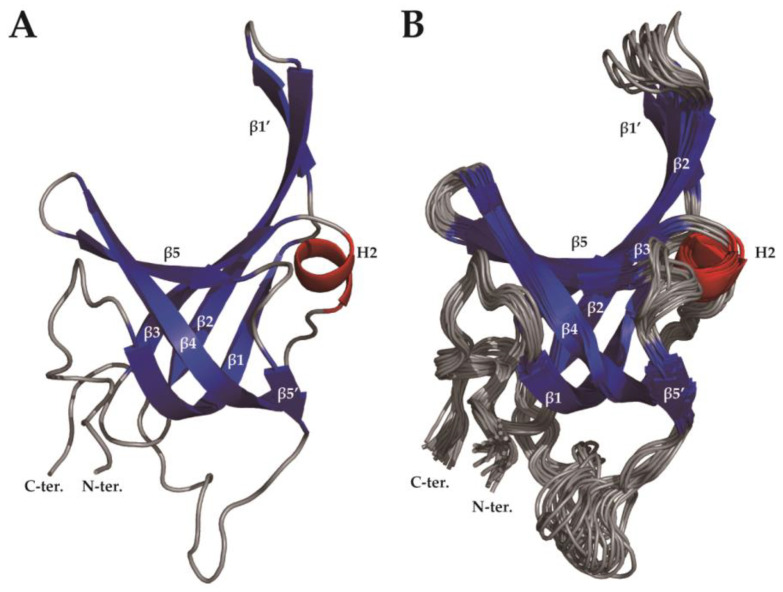
(**A**) The averaged 20 lowest energy solution structures of SsoSSB_1–114_. (**B**) Ensemble of the 20 lowest energy solution structures of SsoSSB_1–114_. The 3_10_ helix is indicated in red, and β strands are shown in blue.

**Figure 4:**
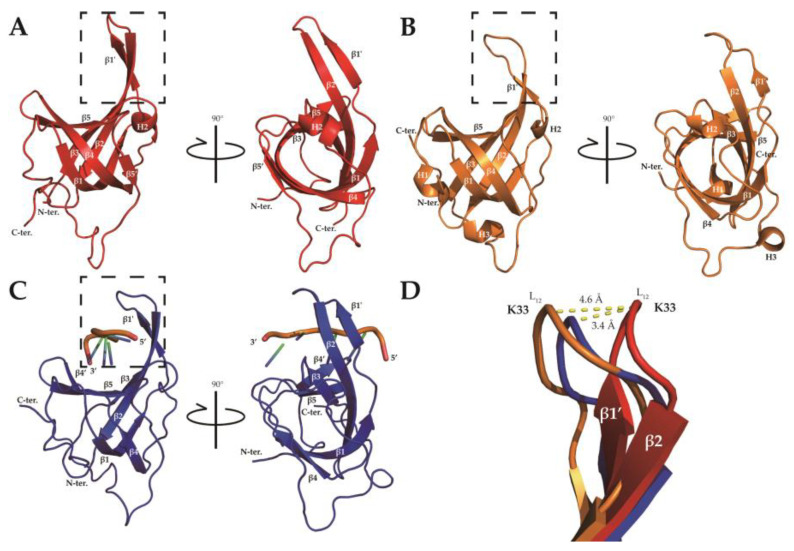
SsoSSB structures by (**A**) NMR at high temperature (PDB ID: 7WCG; indicated with red), (**B**) X-ray crystallography (PDB ID: 1O7I [14]; indicated with orange), and (**C**) NMR at room temperature in complex with ssDNA (PDB ID: 2MNA [15]; indicated with blue). (**D**) Magnified view of the L_12_ loop in each of the 3 structures. The zoomed area is indicated by a dotted square in each figure (**A**–**C**).

The solution structure of the protein was calculated with Xplor-NIH in the PONDEROSA-C/S software package [34], starting from 100 random structures. The structural statistics are shown in Table 1. The 20 lowest energy models (Figure 3) were calculated with no violations and root-mean-square deviations (RMSDs) of 0.974 Å (backbone atoms) and 1.751 Å (heavy atoms). Ramachandran plot analysis from PROCHECK [35] revealed that all dihedral angles were within the allowed regions. The protein retained its secondary and tertiary structure at high temperature, containing five β strands and one 3_10_ helix (Figure 4a and Figure S3). Unstructured regions, especially the L_12_ loop, residues 98–103, and the C-terminal region, were relatively not converged one another. The structure was deposited in the Protein Data Bank (PDB ID: 7WCG).

It was already known that β strands β1, β4, and β5 are broken by residues 26, 72–73, and 89, respectively, which differs from the general OB-fold domain [14]. Secondary structure prediction from the previous study suggested that SsoSSB_1–114_ consists of nine β strands and three 3_10_ helices [28]. The previous X-ray crystal structure (PDB ID: 1O7I) consisted of five β strands and three 3_10_ helices, and the DNA-bound NMR structure at room temperature (25 °C) consisted of five β strands and two 3_10_ helixes (Figure 4b,c and Figure S3). Most β strands were conserved in all structures. There were no 3_10_ helices near the N- and C-termini in our calculated structure, leaving only the internal H2 helix. β strand β5’ was found only in the NMR structures and not in the X-ray structure. It is expected that these differences are partly due to the differences in the structure calculation methods of NMR and X-ray crystallography. The RMSD in the crystal structure (PDB ID: 1O7I) was calculated as 1.406 Å, and that in the solution structure at room temperature bound to ssDNA (PDB ID: 2MNA) was 1.954 Å.

Structural alignment of the three SsoSSB structures revealed that the L_12_ loop in the high-temperature structure of SsoSSB highly deviates from the other two SsoSSB structures. Significant structure fluctuations in L_12_ were also observed in the 20 ensemble structures (Figure 3b), implying that this loop region is highly flexible. This feature is consistent with the previous findings that L_12_ was shown to be flexible from the asymmetric unit superimposition of the X-ray crystal structure [14] and the previous NMR study at room temperature [15]. The position of K33 α-carbon differed by 1.622 ± 0.985 Å (7WCG) and 0.507 ± 0.190 Å (2MNA), respectively. The L_12_ loop, one of the regions forming the DNA binding pocket and that plays an important role in DNA binding of the OB-fold [14,16,17], is less converged at 7WCG than 2MNA. The position of the K33 α-carbon differed by 4.72 ± 1.61 Å (1O7I) and 3.88 ± 1.50 Å (2MNA), respectively. The position of the K33 α-carbon of lowest energy differed by 4.6 Å (1O7I) and 3.4 Å (2MNA), respectively (Figure 4d). L_12_ of the crystal structure was bound to a sulfate ion, whereas L_12_ of the room temperature NMR structure was bound to ssDNA. The high temperature structure confirmed that L_12_ became more flexible and straightened because of the absence of its binding partner, ssDNA.

### 2.3. SsoSSB_1–114_–DNA Interaction at High Temperature by NMR CSP Analysis

**Figure 5:**
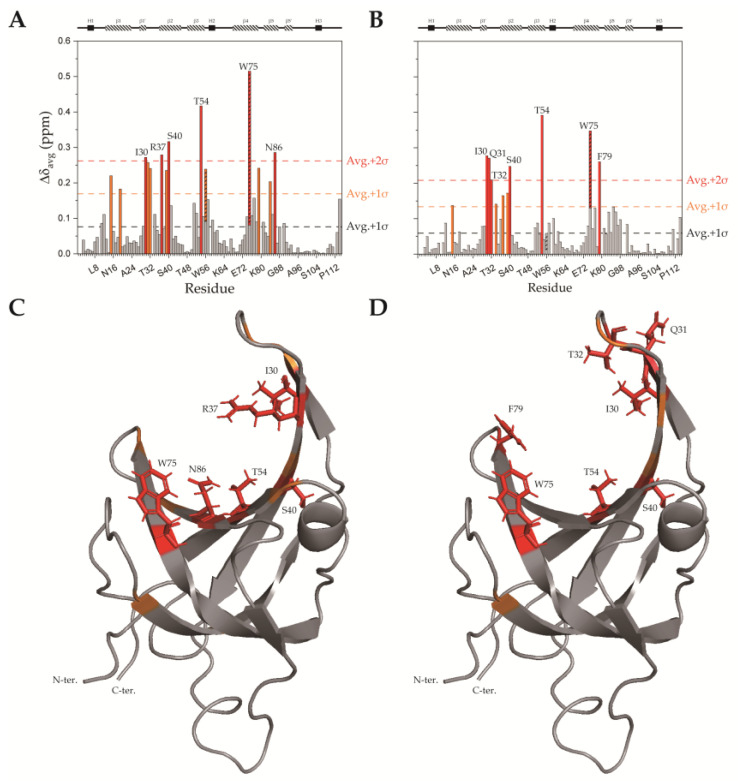
DNA binding site of SsoSSB_1–114_. (**A**,**B**) Chemical shifts of ^15^N-labeled SsoSSB_1–114_ at a concentration of 500 μM were perturbed upon ssDNA titration. Average CSP values (Δδ_avg_) for each residue of SsoSSB_1–114_ with 1 mM ssDNA at (**A**) 25 °C and (**B**) 50 °C are shown. Trp sidechain CSPs are indicated with hatched bars. The secondary structure from UniProt is shown at the top of each graph. (**C**,**D**) The solution structure of SsoSSB_1–114_ (PDB ID: 7WCG) was colored based on CSP data at (**C**) 25 °C and (**D**) 50 °C. Color coding is the same as in panels A and B. Sidechains are displayed for residues with the largest CSPs.

The Gamsjaeger group demonstrated that SsoSSB_1–114_ binds to ssDNA at both room temperature and high temperature [15]. To obtain detailed DNA binding surfaces at the atomic level, we performed chemical shift perturbation (CSP) experiments with ssDNA at 25 °C and 50 °C (Figure 5). The average (standard deviation) CSP during ssDNA titration at 25 °C was 0.0763 (0.0928) ppm, and that at 50 °C was 0.0591 (0.0745) ppm. At 25 °C, residues V15, V19, Q31, T32, I39, W56 (sidechain atoms), F79, and Q84 were perturbed more than 1 standard deviation from the average, and residues I30, R37, S40, T54, W75 (sidechain atoms), and N86 were perturbed more than 2 standard deviations from the average. At 50 °C, residues V15, N34, R37, and I39 were perturbed more than 1 standard deviation from the average, and residues I30, Q31, T32, S40, T54, W75 (sidechain atoms), and F79 were perturbed more than 2 standard deviations from the average. Thus, the ssDNA binding sites and ssDNA binding interactions are similar at 25 °C and 50 °C. At the higher temperature, the perturbation of residues R37, Q84, and N86 was reduced, suggesting that the charged and polar interaction with ssDNA decreases at high temperature, and the hydrophobic interaction mainly remains. In the study from Kerr et al. [14], I30, W56, W75, and F79 were shown by alanine substitution to be important for ssDNA binding at 50 °C. Among these point mutants, the ssDNA binding affinity of W56A was reduced the most, but in our experiments, the sidechain of W56 did not interact with ssDNA at 50 °C (Figure 5b,d). It can be inferred from this that W56 indirectly affects the protein’s ssDNA binding at 50 °C.

### 2.4. Backbone Dynamics of SsoSSB_1–114_ at High Temperature and Room Temperature

**Figure 6:**
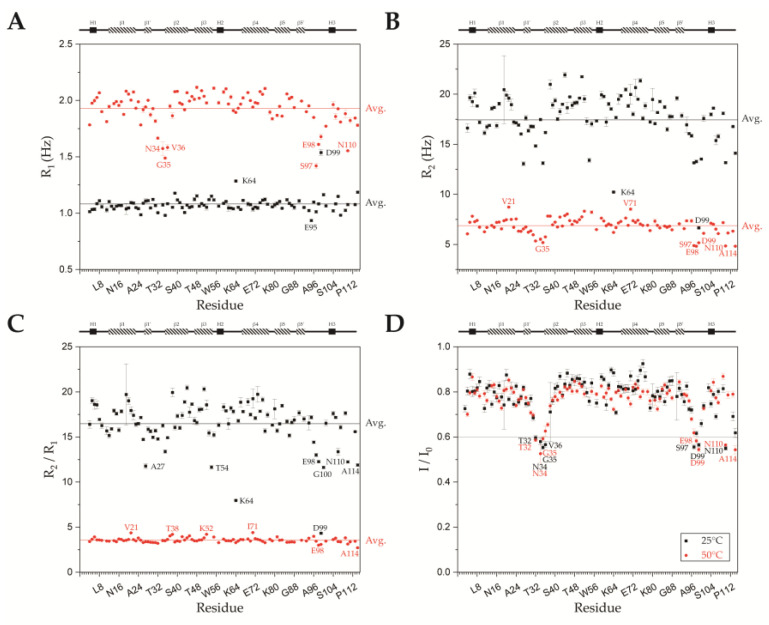
SsoSSB_1–114_ backbone dynamics. Per residue (**A**) spin-lattice relaxation (R_1_), (**B**) spin-spin relaxation (R_2_), (**C**) R_2_/R_1_ ratios, and (**D**) hetNOE values at 25 °C and 50 °C are shown. Errors of the measurement are indicated by black and red lines. In panels A–C, average values of each parameter are indicated with a black (25 °C) or red (50 °C) line. Residues that differed from the average by more than 2 standard deviations are labeled. In panel (**D**), the hetNOE value 0.6 is indicated with a black line. Residues with values lower than 0.6 are labeled in black (25 °C) or red (50 °C). The secondary structure from UniProt is shown at the top of the graph.

To understand how the protein’s backbone dynamics change with temperature, spin-lattice relaxation (R_1_), spin-spin relaxation (R_2_), and ^1^H-^15^N heteronuclear Overhauser effect (hetNOE) experiments were performed at 25 °C and 50 °C. The average R_1_ at 25 °C was 1.078 ± 0.067 Hz. Most residues had R_1_ values within 2 standard deviations of the average. At 50 °C, the average R_1_ value (1.928 ± 0.141 Hz) was higher than that at 25 °C, and we found more deviations. This implies that the overall motion increases at 50 °C. Residues N34, G35, and V36 in loop L_12_; residues S97 and E98 located in the loop between β5’ and H3; residue N110 at the C-terminus had R_1_ values > 2 standard deviations below the average (Figure 6a). Those regions have no secondary structural elements in 7WCG (Figure S3). In the X-ray crystal structure [14], L_12_ and the region containing residues 94–100 were also found to be flexible. It is characteristic that the lower R_1_ values were observed in a flexible region at 50 °C, unlike at 25 °C. Figure 6b shows the measured R_2_ values of each residue at 25 °C and 50 °C. At 25 °C, the average R_2_ value was 17.66 ± 2.42 Hz. Residues in loop L_12_ and the C-terminal region showed reduced R_2_ values, which indicate fast ps-ns dynamics [39]. At 50 °C, the average R_2_ decreased significantly to 6.862 ± 0.797 Hz. This also suggests that the protein becomes more flexible at 50 °C. Residues that had reduced R_1_ values at 50 °C also showed reduced R_2_ values. From the R_1_ and R_2_ data, it was confirmed that the residues that experience fast dynamics were more prominent at 50 °C, and the overall motion of the protein was faster at a higher temperature. From Figure 6c, we can observe higher R_2_/R_1_ values at 25 °C than 50 °C. The average tumbling time (τ_c_) values calculated from R_2_/R_1_ were 12.657 ns and 5.012 ns at 25 °C and 50 °C, respectively [40,41], indicating that the protein tumbles twice as fast at high temperature as it does at room temperature.

From the hetNOE data (Figure 6d), residues T32, N34, G35, and V36 located in L_12_; residues S97 and D99 in the loop between β5 and H3; N110 at the C-terminus had hetNOE values lower than 0.6 at 25 °C. Residues T32, N34, G35, E98, D99, N110, and A114 had hetNOE values lower than 0.6 at 50 °C. Thus, these regions are unstructured and more flexible, consistent with previous studies [14,15] and our R_1_, R_2_ relaxation experiments. The average hetNOE values were 0.780 and 0.766 at 25 °C and 50 °C, respectively, showing that the overall rigidity of the protein is maintained at 50 °C. Unlike the R_1_ and R_2_ values, the hetNOE values did not show a significant difference by temperature. Because the hetNOE value reflects the motion within the protein rather than the global motion of the protein within its chemical environment, this suggests that the internal motion of the protein is not changed much at increased temperatures [42].

### 2.5. Thermostability and ssDNA Binding Property of SsoSSB_12–114_

**Figure 7:**
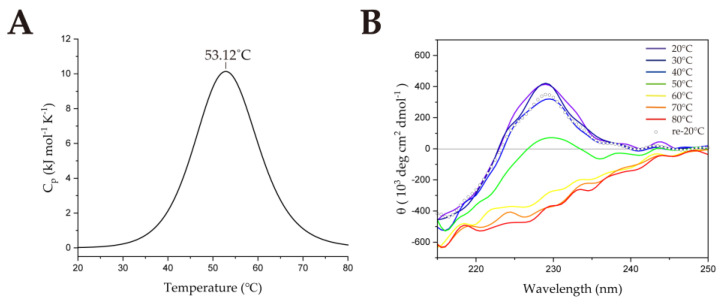
Thermostability of SsoSSB_12–114_. (**A**) Melting temperature of SsoSSB_12–114_ measured by differential scanning calorimetry. (**B**) Circular dichroism spectroscopy was used to investigate the effect of temperature on the secondary structure of SsoSSB_12–114_.

It was already known that the helix between β3 and β4 in the OB-fold family is well conserved and makes a significant contribution to structural stabilization [17]. However, studies on the importance of the helix near the N-terminus are lacking. Because the N-terminal region of SsoSSB is not conserved among bacterial SSBs (Figure 1) and TmaSSB lacking this region has higher T_m_ [22], we hypothesized the N-terminal region of SsoSSB is not crucial for the structural stabilization. The structure of SsoSSB (PDB ID: 7WCG) and TmaSSB (PDB ID: 1Z9F [43]) ss shown in the Supplementary Materials (Figure S4). To investigate the role of the N-terminal region, an N-terminal deletion mutant (SsoSSB_12–114_) was prepared. DSC was used to measure the T_m_ value of SsoSSB_12–114_ as 53.12 °C (Figure 7a). This was ~30 °C lower than the T_m_ value of SsoSSB_1–114_ and in a similar range to the mesophilic SSB, hRPA (70A subunit; 56.69 °C, Figure S5). CD spectroscopy was performed at 20 °C to confirm the secondary structure of the protein (Figure S1a). The overall pattern was very similar to SsoSSB_1–114_, except that lower molar ellipticities were observed at 215 nm and 228 nm. The molar ellipticity was not fully recovered after heating and cooling (Figure 7b). Unlike the SsoSSB_1–114_, molar ellipticity at 228 nm also changed significantly between 50 °C and 60 °C (Figure S1b). This is consistent with the DSC data. Together, these findings imply that SsoSSB_12–114_ entirely loses thermostability. To monitor the structural changes that occur upon deletion of the N-terminal helix, we performed an ^1^H-^15^N HSQC experiment on SsoSSB_12–114_ at 25 °C (Figure S6a). The NMR spectra showed that the structure was not disordered, but when comparing the ^1^H-^15^N HSQCs of SsoSSB_1–114_ and SsoSSB_12–114_, we observed that more than half of the peaks shifted due to the N-terminal deletion (Figure S6b). 

A DNA titration was performed to see if SsoSSB_12–114_ could still interact with ssDNA. There were some chemical shift changes observed due to the added ssDNA (Figure S6c). ITC experiments were also performed to measure the binding affinity. The dissociation constant (K_d_) and stoichiometry (n) of SsoSSB_1–114_ in the presence of dA(15) were 1.75 μM and 1.009 (Figure S7a), respectively, but we did not observe enough heat from the interaction of SsoSSB_12–114_ and dA(15) to determine thermodynamic parameters (Figure S7b). Taken together, the deletion of the N-terminal 11 amino acids from SsoSSB dramatically affected its thermostability, structure, and DNA binding capability.

## 3. Discussion

In this study, we investigated the solution structure, DNA binding properties, and dynamic properties of the thermophilic SsoSSB at high temperature (50 °C). While the protein contains a well-conserved OB-fold domain and its structural aspects were already studied at room temperature [14,15], the structural and dynamic origins of thermophilicity were still not clearly understood. In this study, we collected NMR data to analyze the structure and backbone dynamics at 50 °C. We believe that this approach provides unique information to understand this thermophilic protein. 

It is usually considered that a sufficient number of NOE (i.e., 10–20 NOEs per residue) are required for the reliable protein NMR structure calculation [44]. However, a recent study showed that the restraints per residue do not guarantee the accuracy of the structure. At the same time, Ramachandran analysis could be considered the accuracy indicator of the NMR structures [45]. While we used a relatively low number of NOE on average, we could collect a substantial number of long-range NOE using AUDANA algorithms for the structure calculation. The structural statistics (Table 1) showed that our structure is acceptable and reflects the protein’s nature. 

**Figure 8:**
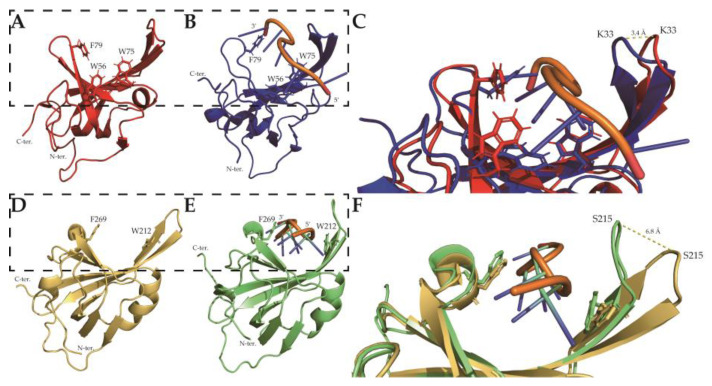
Protein structures of OB-fold proteins apo form and ssDNA bound form. NMR structure of SsoSSB (**A**) apo form (PDB ID: 7WCG; indicated with red) and (**B**) with ssDNA (PDB ID: 2MNA [15]; indicated with blue). (**C**) Zoom of the structure overlapping of (**A**,**B**). X-ray structure of hRPA70A (**D**) apo form (PDB ID: 1FGU [46]; indicated with yellow-orange) and (**E**) with ssDNA (PDB ID: 1JMC [47]; indicated with lime). (**F**) Zoom of the structure overlapping of (**D**,**E**). Aromatic residues which interact with ssDNA show with sidechain stick structure. The zoomed areas are indicated by a dotted square in each figure.

Overall, our solution structure was similar to the previously described structures, while local differences in L_12_ were revealed (Figure 8a–c). It is assumed that these differences were caused by the conformational change upon ssDNA binding, and that the flexibility of the region could contribute to the differences. Similar conformational differences were observed in hRPA70A, a eukaryotic OB-fold protein. The X-ray crystal structure of the apo form of hRPA70A (PDB ID: 1FGU [46]) and the ssDNA bound form of hRPA70A (PDB ID: 1JMC [47]) showed that the L_12_ gets closer to the DNA and has a ‘closed’ conformation in the presence of ssDNA (Figure 8d–f). In the apo versus the DNA-bound form, the α carbon of S215, located at the top of L_12_, shifts by 6.8 Å (Figure 8f), which is larger than the equivalent difference in SsoSSB (3.4 Å). In this regard, we suggest that our solution structure at high temperature represents the apo form of SsoSSB under near physiological conditions.

The aromatic residues involved in the stacking interaction with the DNA are shown in Figure 8a,b. Even though W56 is well conserved in bacterial SSBs (Figure 1), the structure showed that it contributes less than the other two residues (W75 and F79). This structure is consistent with our CSP analyses (Figure 5). Furthermore, we observed subtle differences in the DNA binding interface depending on the temperature. More electrostatic interactions were involved at room temperature, while hydrophobic interactions were more crucial at high temperature. This suggests that the nonspecific DNA binding of SsoSSB is mediated by an optimal combination of noncovalent interactions depending on the environment. 

Because protein backbone dynamics are not usually assessed at a high temperature, it is not easy to compare our data with others. At or near room temperature, regions with ps-ns dynamics usually have higher R_1_ (reduced T_1_) and lower R_2_ (elevated T_2_) values [40]. At 50 °C, we observed that the average R_1_ increased and R_2_ decreased compared to the values at 25 °C (Figure 6a,b). This could reflect the general physical phenomena of proteins: the overall motion increases with elevated temperature. This interpretation is consistent with the rotational correlation time at 50 °C, being shorter than that at 25 °C. Unlike at 25 °C, the per residue R_1_ value at 50 °C showed that the flexible regions such as the L_12_ loop and the C-terminus have lower R_1_ value than average (Figure 6a). This might be related to the shortened rotational correlation time at 50 °C. One of the possible explanations is that the R_1_ of the flexible region decreases in the same way as R_2_ under conditions where τ_c_ is faster than the value expected from the protein’s molecular weight [48]. Remarkably, the per residue hetNOE values were similar at both temperatures. Our data clearly showed that the overall protein folding was well maintained even at 50 °C, consistent with our DSC and CD data.

Previous studies found that electrostatic and hydrophobic interactions play an essential role in the thermal stabilization of thermophilic proteins [49,50,51,52,53]. We discovered that the N-terminus (residues 1 to 11) of SsoSSB is important for maintaining thermostability, even though it is located at the terminus of the protein and is not conserved across bacterial SSBs. An N-terminal deletion caused T_m_ to decrease ~30 °C. The absence of the N-terminus resulted in partial destabilization of the protein (Figure 7b), affecting thermostability (Figure 7a) and DNA binding interaction (Figure S7b). These large disruptions led us to speculate that the N-terminus might contribute to the stability of the protein by acting as the lid of the β-barrel of the OB-fold. Since the binding affinity to ssDNA was so significantly reduced due to the absence of the N-terminal sequence (Figure S7b), which does not directly interact with ssDNA, it is reasonable to propose that the presence of the N-terminus is essential for maintaining the tertiary structure. From point mutation studies of the *Thermotoga maritima* acyl carrier protein [49], the T_m_ decreases significantly by removing particular noncovalent interactions, while the mutant had a similar structure. Our study showed a different way to modulate the thermostability of these proteins, namely by truncating a region that is not included in the core structure. Further studies of the deletion mutant and other point mutants are required to reveal the complete origins of the thermostability of SsoSSB.

Based on our current understanding of the important residues and regions for thermostability, it is expected that it will be possible to make proteins with improved thermostability. For decades, efforts to improve protein stability and thermostability by protein engineering have continued. Introducing new disulfide bonds [54,55], optimizing metal chelation sites [56], and amino acid substitutions [57,58] have been thoroughly researched. However, improving thermal stability requires a lot of time and money. Recently, several computational studies [59,60] have been used to overcome these difficulties in biological research. In addition, deep learning and machine learning techniques have been employed to improve protein stability and thermostability [60,61,62]. Our study provides detailed information on the structure and ssDNA interactions of SsoSSB at high temperature. This information can provide fundamental insights into SSoSSB’s industrial applications, such as increasing polymerase chain reaction efficiency [63], detecting viral nucleic acid [64], and potentially increasing the stability of mRNA vaccines [65].

## 4. Materials and Methods

### 4.1. Protein Expression and Purification

SsoSSB_1–114_ and SsoSSB_12–114_ were cloned into a pET C-terminal TEV His6 cloning vector with BioBrick polycistronic restriction sites (9Bc) and transformed into BL21(DE3) cells. We cultivated cells for more than 12 h in 10 mL LB medium (25 g/L) with ampicillin (0.3 mM, final concentration) at 37 °C. Into 1 L of LB medium containing ampicillin, 15 mL of overnight cultured cells was poured. Cells were grown at 37 °C until the optical density at 600 nm reached 0.5–0.6, and then isopropyl β-D-1-thiogalactopyranoside was added to a final concentration of 0.5 mM. Cells were incubated for an additional 14–18 h at 18 °C. Cells were centrifuged for 15 min at 7500 rpm at 4 °C. For separating endogenous nucleic acids from protein, we used a high salt binding buffer (50 mM NaH_2_PO_4_, 2 M NaCl, pH 8.0) and a high salt wash buffer (50 mM NaH_2_PO_4_, 2 M NaCl, 40 mM imidazole, pH 8.0). Cells were resuspended and sonicated in the high salt binding buffer. The sample was centrifuged for 15 min at 13,000 rpm, 4 °C, and the supernatant put into an Ni-NTA column (Cytiva, Marlborough, MA, USA). The high salt wash buffer and an elution buffer (50 mM NaH_2_PO_4_, 300 mM NaCl, 300 mM imidazole pH 8.0) were used sequentially to purify the proteins. The proteins were further purified by gel filtration chromatography using a Hi-Load 16/600 75 pg column (Cytiva, Marlborough, MA, USA) with buffer A (100 mM NaCl, 20 mM 2-(N-morpholino)ethanesulfonic acid (pH 6.5). For expression of ^15^N- and ^13^C-labeled protein, cells were grown in M9 minimum media that included ^15^NH_4_Cl and ^13^C-D-Glucose (Cambridge Isotope Laboratories, Inc., Tewksbury, MA, USA) as nitrogen and carbon sources. The composition of M9 minimum media was 870 mL of distilled water, 1 g of ^15^NH_4_Cl, 100 mL of M9 10X salt, 20 mL of 10% glucose (^13^C-labeled) solution, 2 mL of 1 M MgSO_4_ solution, 0.3 mL of 1 M CaCl_2_ solution, 0.33 mL of vitamin solution, and 10 mL of trace metal solution.

### 4.2. NMR Experiments 

The ^15^N- and ^13^C-labeled SsoSSB_1–114_ sample was dissolved to a final protein concentration of 0.5–1.2 mM with 10% D_2_O in buffer A. A Bruker 900 MHz NMR spectrometer equipped with a cryogenic triple-resonance probe at the Korea Basic Science Institute (Ochang, Korea), Bruker AVANCE Neo 600 MHz spectrometers at GIST Central Research Facilities with a cryogenic triple-resonance probe (Gwangju, Korea), and an Agilent DD2 700 MHz NMR spectrometer at Gyeongsang National University (Jinju, Korea) were used to collect NMR spectra. Backbone and sidechain assignments were performed in previous studies [28,66]. ^15^N- and ^13^C-edited NOESY-HSQC were collected at 50 °C with 150 ms and 300 ms mixing times for structure calculation. In CSP experiments, ssDNA composed of 15 adenines (dA(15)) was added at molar ratios ranging from 0:1 to 2:1 to ^15^N-labeled SsoSSB_1–114_. Average CSP values (Δδ_avg_) were calculated using the following equation

(1) $${\Delta \delta }_{\mathrm{avg}}=\sqrt{{\left(\frac{\Delta \delta N}{5.88}\right)}^{2}+{\left(\Delta \delta H\right)}^{2}}$$

### 4.3. Solution Structure Calculation 

The 3D structure of SsoSSB_1–114_ at 50 °C was calculated using Xplore-NIH-based computations in the PONDEROSA-C/S package [35], and NOE assignments were performed using NMRFAM-Sparky [37]. Following that, the 20 lowest energy structures were determined. PONDEROSA-Analyzer software [67] was used to assess and refine all angle and distance violations of the best 20 constructions. PSVS [36] was used to analyze the final 20 lowest energy structures. PyMOL (http://www.pymol.org, accessed on 8 March 2022) was used to create the protein structural diagrams and align the protein structures. The NOE constraints and final coordinates were deposited in the RCSB PDB under the accession number 7WCG (BMRB ID: 50523).

### 4.4. NMR Backbone Relaxation Experiment

R_1_ and R_2_ of ^15^N, and ^1^H-^15^N hetNOE data, were recorded on the Bruker AVANCE Neo 600 MHz spectrometers at GIST Central Research Facilities with cryogenic triple-resonance probes (Gwangju, Korea). Pseudo-3D NMR spectra were collected with relaxation delays of 20, 60, 100, 200, 400, 600, 800, 1000, 1200, and 1600 ms at 25 °C and 50 °C for the ^15^N R_1_ measurements, and with relaxation delays of 16.96, 33.92, 67.84, 101.76, 135.68, 203.52, 271.36, 339.2, 407.04, and 547.72 ms at 25 °C and 50 °C for the ^15^N R_2_ measurements. POKY was used to extract the relaxation rate constants by fitting the decay of peak height as a function of the relaxation delay to a single exponential function [38]. For the hetNOE measurement, interleaved 2D ^1^H-^15^N HSQC spectra were acquired with and without an initial proton saturation of 2.5 s at 25 °C and 50 °C. hetNOE values were obtained from the ratios of peak heights between pairs of spectra, calculated with a POKY script [38]. For more accurate analysis, overlapping peaks were excluded from the data. The rotational correlation time (τ_c_) was calculated by this equation [39,40]

(2) $$\tau_{c}=\left(\frac{1}{4{\pi \nu }_{N}}\right)\sqrt{\left(6\frac{R_{2}}{R_{1}}-7\right)}$$

where ν*_N_* is the resonance frequency of ^15^N in Hz.

### 4.5. Differential Scanning Calorimetry

The T_m_s of SsoSSB_1–114_, SsoSSB_12–114_, and hRPA70A were measured by DSC using a NanoDSC system (TA instruments, New Castle, DE, USA). The protein samples were prepared at concentrations of 5 mg/mL in buffer A. The thermograms were recorded as the temperature was increased at a rate of 1 °C/min from 50 °C to 110 °C (SsoSSB_1–114_) or 20 °C to 80 °C (SsoSSB_12–114_ and hRPA70A). The pressure was kept constant at 3 atm to prevent evaporation of the solvent. Individual component peaks were resolved from the complex profiles after polynomial baseline correction, and the two-state scaled curve fittings were performed by the NanoAnalyze software (TA Instrument, New Castle, DE, USA).

### 4.6. Circular Dichroism Spectroscopy 

The secondary structure of SsoSSB at various temperatures was assessed by far-UV CD experiments using a J-815 spectropolarimeter (Jasco, Tokyo, Japan). It was measured under two different conditions. A 100 μM protein sample was dissolved in buffer B (20 mM NaHPO_4_, pH 6.5) and placed in a cuvette with a 0.2 mm path length. CD spectra were measured from 190 to 250 nm at 0.5 nm intervals at 20 °C. A 50 μM protein sample was dissolved in buffer A and placed in a cuvette with a 1 mm path length. CD spectra were measured from 210 to 250 nm at 0.5 nm intervals. The temperature was increased from 20 to 80 °C in 2 °C increments. After heating, the temperatures were decreased from 80 °C to 20 °C in 5 °C decrements. Every measurement was performed after waiting for 1 min between temperature changes. Temperature-dependent ellipticity changes at 228 nm were observed to monitor the heat denaturation of the protein. θ was calculated as described in previous papers [23,25,26,27].

### 4.7. Isothermal Titration Calorimetry

ITC experiments were carried out in buffer A with a Nano-ITC SV instrument (TA Instruments, New Castle, DE, USA). Twenty-four aliquots of 10 μL of 500 μM dA(15) were titrated at 25 °C into 50 μM of SsoSSB_1–114_ and SsoSSB_12–114_. The stirring speed was 300 rpm, and the interval between titrations was 250 s. The dissociation constant (K_d_) and stoichiometry (n) were calculated by fitting to the independent model in the NanoAnalyze software (TA Instruments, New Castle, DE, USA).

## Figures and Tables

**Table 1:** Statistics of the solution structure of SsoSSB_1–114_ at 50 °C. The 20 lowest energy structures were calculated using NMR restraints.

Restraints ^1^			Value
**Total NMR Constraints**			**1086**
	Distance Constraints		
		Intra Residue (|i–j| = 0)	259
		Sequential Residue (|i–j| = 1)	225
		Medium Range (1 < |i–j| ≤ 5)	85
		Long Range (|i–j| > 5)	291
		Hydrogen Bond	33
	Dihedral Angle Constraints		
		Φ	96
		Ψ	97
**Pairwise RMSD (** **Å) ^2^**			
	Backbone Atoms ^3^		0.974 ± 0.044
	Heavy Atoms ^3^		1.751 ± 0.054
**Ramachandran Plot Summary from PROCHECK (%) ^2^**			
	Most Favored Regions		94.9
	Additionally Allowed Regions		3.9
	Generously Allowed Regions		1.2
	Disallowed Regions		0.0
**wwPDB NMR Structure Validation ^4^**			
	Clashscore		8
	Ramachandran Outliers		2.0%
	Sidechain Outliers		1.0%
**Average Number of Violations Per Conformer ^5^**			
	Distance Violations (>0.5 Å)		0
	Angle Violations (>5°)		0
	Repulsive Violations		0

^1^ The solution structure of SsoSSB_1–114_ was calculated using Xplor-NIH in PONDEROSA-C/S [36]. ^2^ The final 20 lowest energy structures were evaluated using Protein Structure Validation Software (PSVS) [35]. ^3^ Among ordered residues: E3-S97, S104-T113. ^4^ wwPDB (7WCG) validation results [37]. ^5^ Xplor-NIH pseudo-potential energy and every violation of the 20 best structures were analyzed using POKY-Analyzer [38].

## Data Availability

The data presented in this study are available on request from the corresponding author.

## Supplementary Materials

The following supporting information can be downloaded at: https://www.mdpi.com/article/10.3390/ijms23063099/s1.

## Abbreviations

CSP	chemical shift perturbation
CD	circular dichroism
DSC	differential scanning calorimetry
dA(15)	single-stranded DNA composed of 15 adenines
dsDNA	double-stranded DNA
hetNOE	heteronuclear Overhauser Effect
HSQC	heteronuclear single-quantum coherence
ITC	isothermal titration calorimetry
NMR	nuclear magnetic resonance
NOE	nuclear Overhauser effect
OB-fold	oligonucleotide/oligosaccharide binding fold domain
PDB	protein data bank
RMSD	root-mean-square deviation
R_1_	spin-lattice relaxation
R_2_	spin-spin relaxation
SSB	single-stranded DNA binding protein
ssDNA	single-stranded DNA
Sso	*Sulfolobus solfataricus*
T_m_	melting temperature
