# Supplementary material for: NMR Structure and Biophysical Characterization of Thermophilic Single-Stranded DNA Binding Protein from Sulfolobus Solfataricus
Source: Int J Mol Sci. 2022 Mar 13;23(6):3099. doi: 10.3390/ijms23063099 (PMC8954794; doi:10.3390/ijms23063099)
Supplement: Supplementary file 1 [file ijms-23-03099-s001.zip › ijms-1594311-supplementary.pdf]

Supplementary Materials

NMR Structure and Biophysical Characterization of Thermophilic Single-stranded DNA Binding Protein from *Sulfolobus solfataricus*

Min June Yang[1], Jinwoo Kim[1], Yeongjoon Lee[2], Woonghee Lee[2,*], and Chin-Ju Park[1,*]

[1] Department of Chemistry, Gwangju Institute of Science and Technology, Gwangju 61005, Korea; minjune1590@gm.gist.ac.kr (M.J.Y.); wlsdn8810@gist.ac.kr (J.K.)
[2] Department of Chemistry, University of Colorado Denver, Denver, CO 80217-3364, USA; bioyj1012@gmail.com
* Correspondence: woonghee.lee@ucdenver.edu (W.L.); cjpark@gist.ac.kr (C.-J.P.); Tel.: +1-303-315-7672 (W.L.); +82-62-715-3630 (C.-J.P.)

Figure S1: Results of CD spectroscopy. (A) θ value at 190 to 250 nm from CD spectroscopy of $SsoSSB_{1-114}$ and $SsoSSB_{12-114}$ at 20°C. (B) Molar ellipticity at 228 nm of $SsoSSB_{1-114}$ and $SsoSSB_{12-114}$ from 20 to 80°C.

Figure S2: Number of distance constraints used for structure calculation. The number of Short-range (Intramolecular and sequential residue) constraints (white), mid-range constraints (cyan), and long-range constraints (red) are marked. The secondary structure from Uniprot showed the top of the graph.

Figure S3: Secondary structure comparison among the high-temperature NMR structure (PDB ID: 7WCG), the X-ray crystal structure (PDB ID: 1O7I), and the room temperature NMR structure in complex with ssDNA (PDB ID: 2MNA). Aromatic residues which are related to DNA binding are indicated in red.

Figure S4: (A) The lowest energy structure of $SsoSSB_{1-114}$ (PDB ID: 7WCG; The N-terminal part (residue number 1-11) is shown in magenta). (B) The crystal structure of TmaSSB (PDB ID: 1Z9F).,

Figure S5: T_m of hRPA70A measured by DSC.

Figure S6: (A) ^{1}H-^{15}N HSQC of $SsoSSB_{12-114}$. (B) Overlay of ^{1}H-^{15}N HSQC spectra of $SsoSSB_{1-114}$ (blue) and $SsoSSB_{12-114}$ (red). (C) Overlay of ^{1}H-^{15}N HSQC spectra of $SsoSSB_{12-114}$ with ssDNA dA(15) (green), $SsoSSB_{1-114}$ (blue), and $SsoSSB_{12-114}$ (red). Experiments were performed at 25°C.

Figure S7: Thermodynamic analysis by isothermal titration calorimetry. ssDNA was added to (A) $SsoSSB_{1-114}$ and (B) $SsoSSB_{12-114}$. The fitted curve represents a 1 to 1 binding model with the given K_d and n.

Figure S1

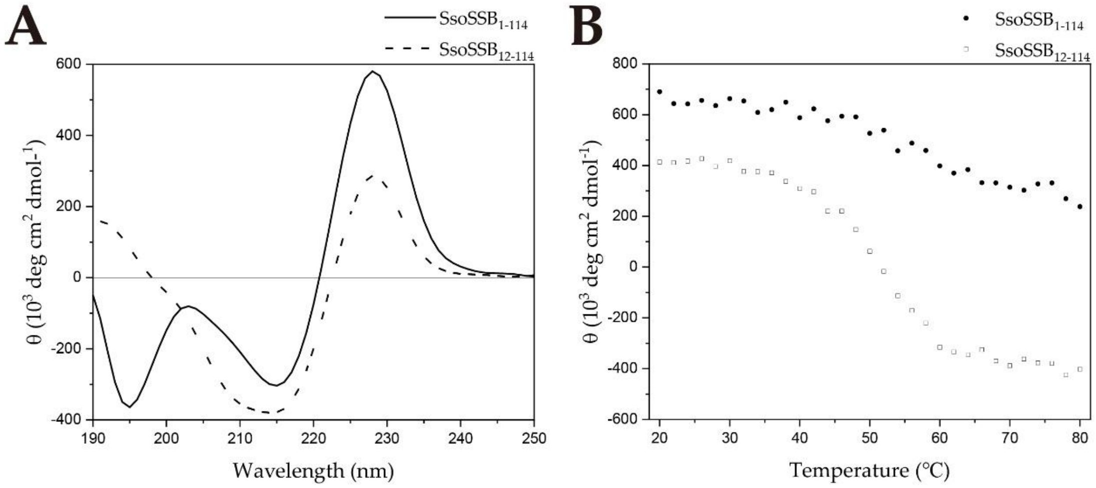
A
SsoSSB1-114
SsoSSB12-114
θ (10³ deg cm² dmol-¹)
Wavelength (nm)
B
SsoSSB1-114
SsoSSB12-114
θ (10³ deg cm² dmol-¹)
Temperature (℃)

Figure S2

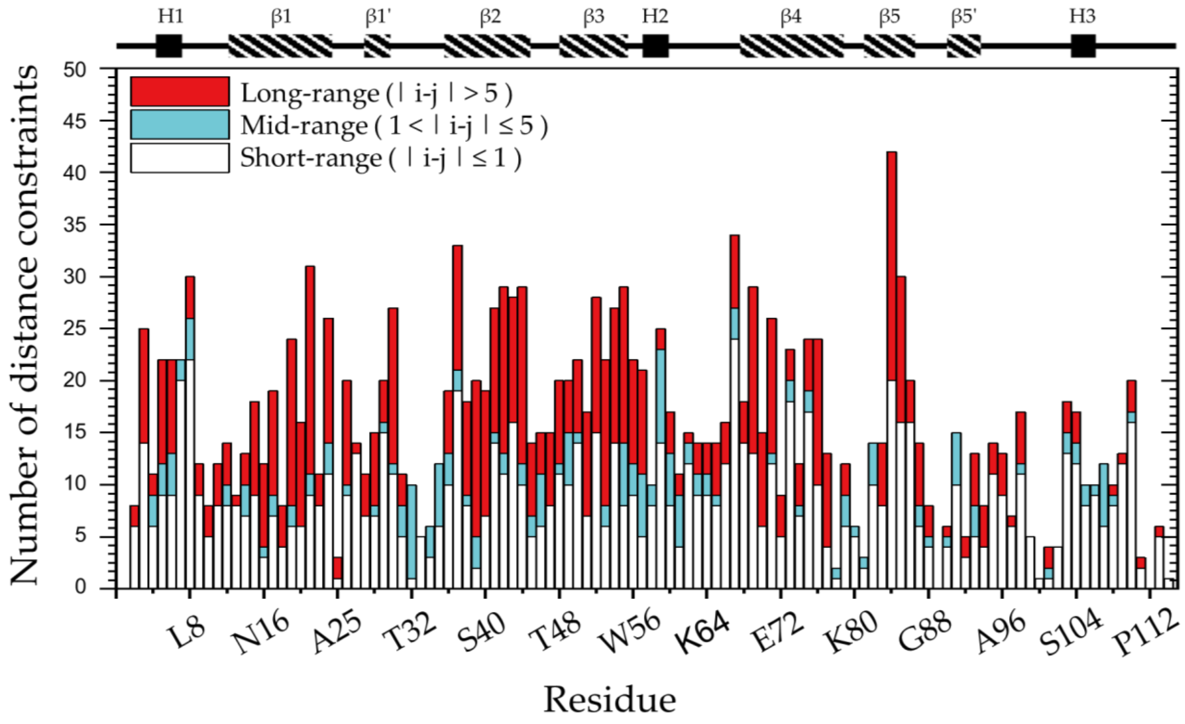

H1
β1
β1'
β2
β3
H2
β4
β5
β5'
H3
Long-range (| i-j | > 5)
Mid-range (1 < | i-j | ≤ 5)
Short-range (| i-j | ≤ 1)
Number of distance constraints
0
5
10
15
20
25
30
35
40
45
50
L8
N16
A25
T32
S40
T48
W56
K64
E72
K80
G88
A96
S104
P112
Residue

Figure S3

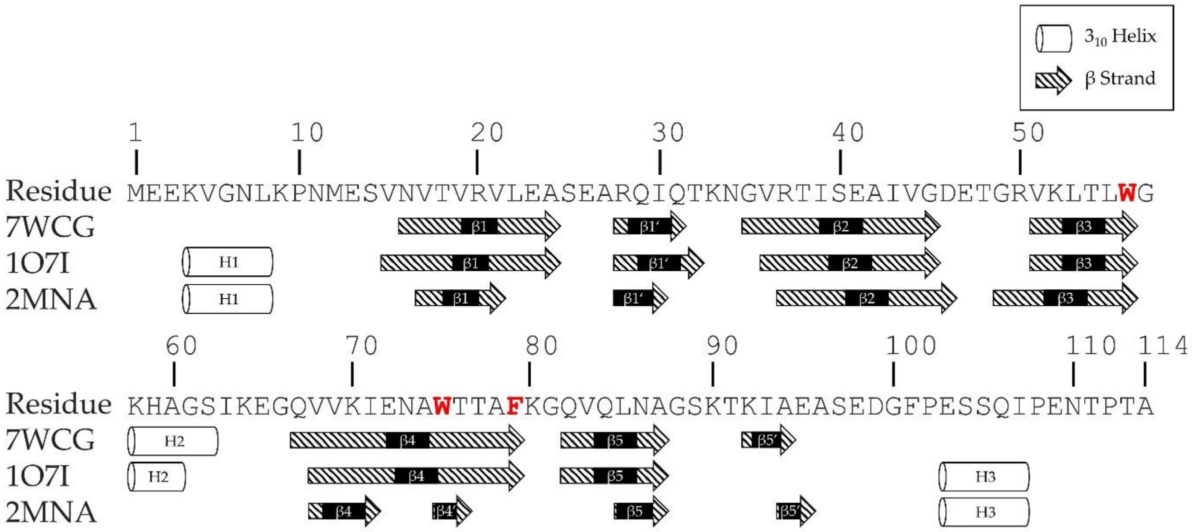

3₁₀ Helix
β Strand
1 10 20 30 40 50
Residue MEEKVGNLKPNMESVNVTVRVLEASEARQIQTKNGVRTISEAIVGDETGRVKLTLWG
7WCG β1 β1′ β2 β3
1O7I H1 β1 β1′ β2 β3
2MNA H1 β1 β1′ β2 β3
60 70 80 90 100 110 114
Residue KHAGSIKEGQVVKIENAWTTAFKGQVQLNAGSKTKIAEASEDGFPESSQIPENTPTA
7WCG H2 β4 β5 β5′
1O7I H2 β4 β5 H3
2MNA β4 β4′ β5 β5′ H3

Figure S4

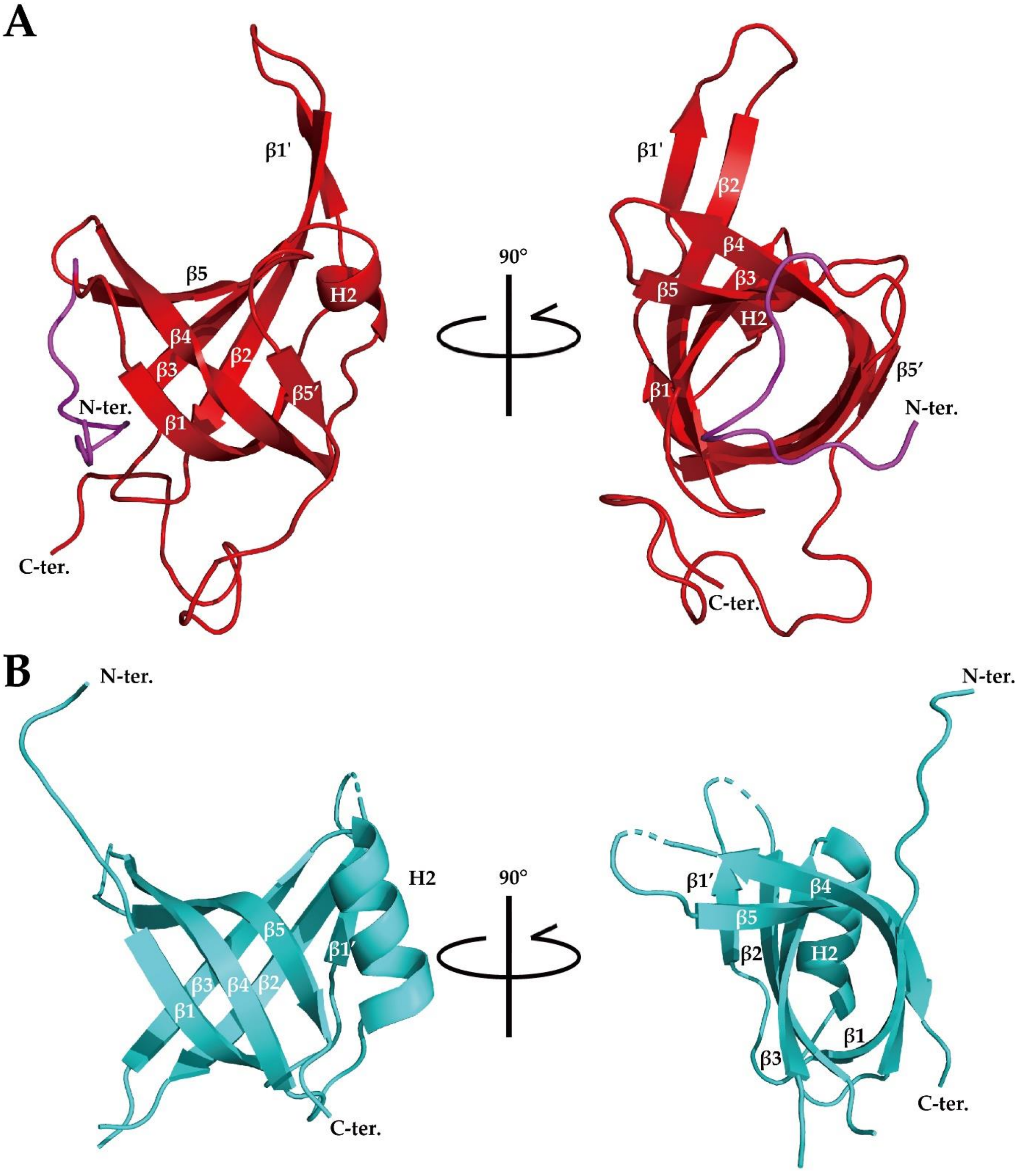
A
β1'
β5
H2
β4
β2
β3
β5′
N-ter.
β1
C-ter.
90°
β1'
β2
β4
β3
β5
H2
β1
β5′
N-ter.
C-ter.
B
N-ter.
H2
β5
β1′
β3
β4
β2
β1
C-ter.
90°
N-ter.
β1′
β4
β5
β2
H2
β1
β3
C-ter.

Figure S5

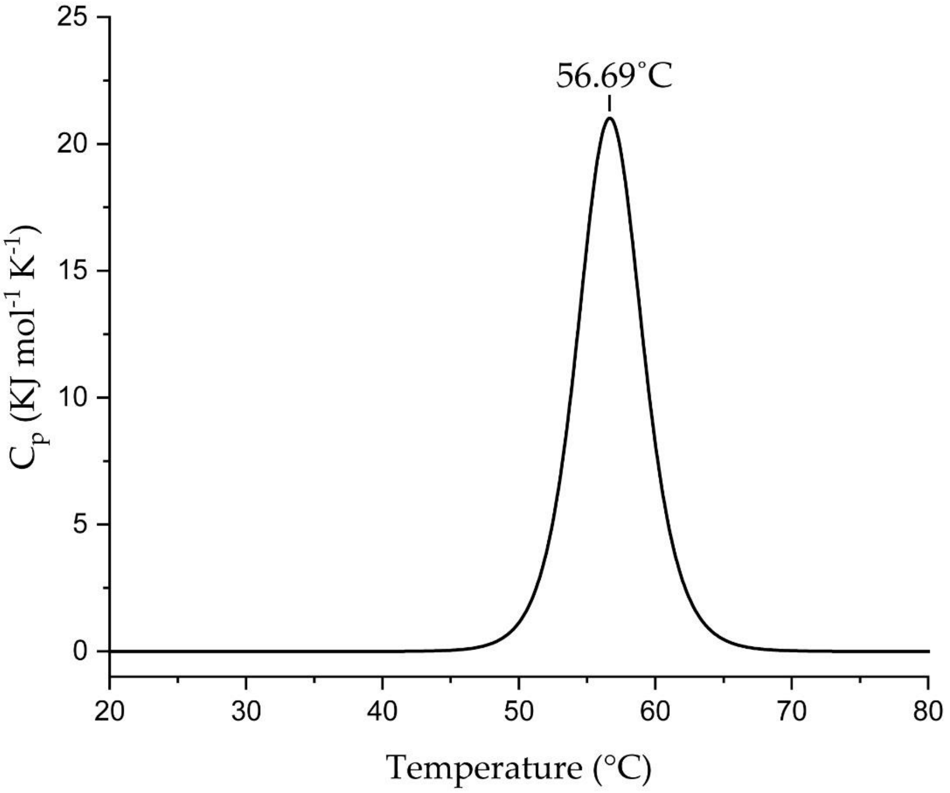

56.69˚C
25
20
15
10
5
0
20
30
40
50
60
70
80
Cp (KJ mol-1 K-1)
Temperature (°C)

Figure S6

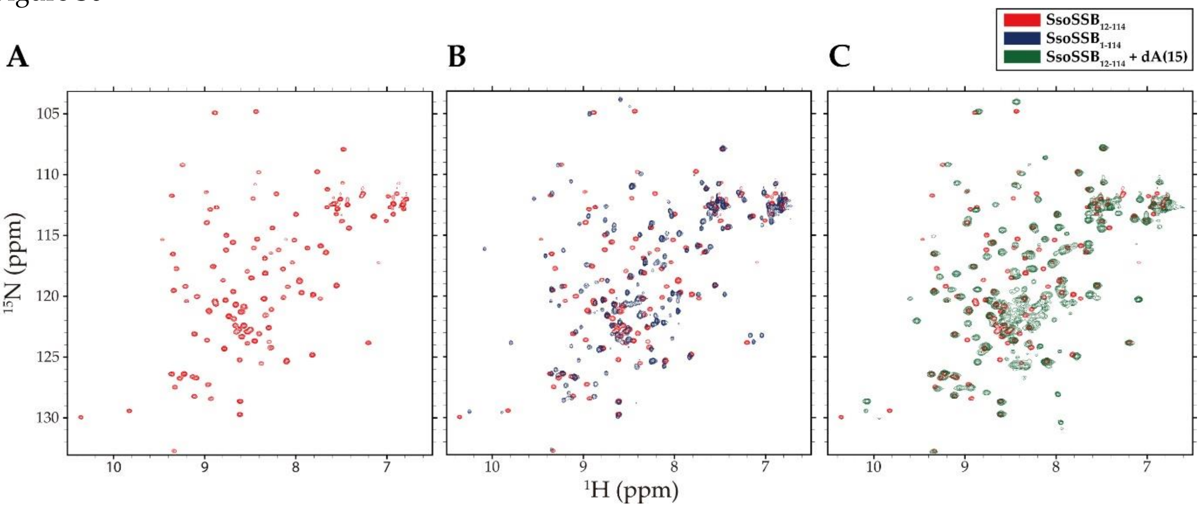
SsoSSB12-114
SsoSSB1-114
SsoSSB12-114 + dA(15)
A
B
C
15N (ppm)
1H (ppm)
105
110
115
120
125
130
10
9
8
7

Figure S7

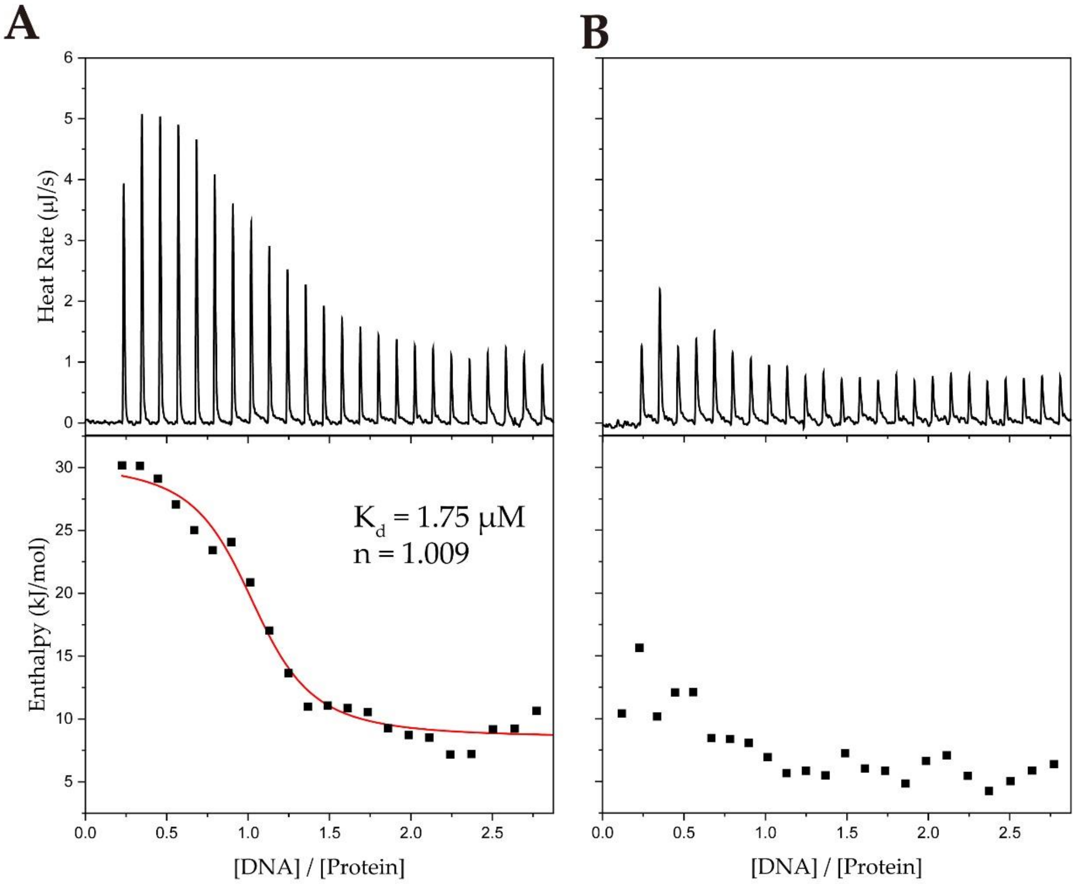

A
B
Heat Rate (μJ/s)
6
5
4
3
2
1
0
30
25
20
15
10
5
Enthalpy (kJ/mol)
K$_d$ = 1.75 μM
n = 1.009
0.0
0.5
1.0
1.5
2.0
2.5
[DNA] / [Protein]
[DNA] / [Protein]
